# COMPREHENSIVE MOBILITY FRAMEWORK VERIFICATION USING CANADIAN LONGITUDINAL STUDY ON AGING DATA

**DOI:** 10.1093/geroni/igad104.3049

**Published:** 2023-12-21

**Authors:** Sandra Webber, Yixiu Liu, Ruth Barclay, Depeng Jiang, Jacquie Ripat, Scott Nowicki, Robert Tate

**Affiliations:** University of Manitoba, Winnipeg, Manitoba, Canada; University of Manitoba, Winnipeg, Manitoba, Canada; University of Manitoba, Winnipeg, Manitoba, Canada; University of Manitoba, Winnipeg, Manitoba, Canada; University of Manitoba, Winnipeg, Manitoba, Canada; University of Manitoba, Winnipeg, Manitoba, Canada; University of Manitoba, Winnipeg, Manitoba, Canada

## Abstract

Mobility within and between life spaces (one’s room, home, local community, and further destinations) positively influences quality of life and health in older adults. Understanding factors that impact mobility is relevant in many contexts (e.g., in clinical and research environments, for city-planning and social programming). The purpose of this study was to verify a comprehensive theoretical framework for mobility (Webber et al., 2010) with structural equation modelling using data from the Canadian Longitudinal Study on Aging (CLSA). We estimated associations between latent factors consisting of physical, psychosocial, environmental, financial, and cognitive attributes, and life space mobility for participants 65-85 years of age (n=11,667, mean age 73 ± 6 years) with age, sex and education as covariates. The model demonstrated good fit (CFI = 0.90, RMSEA (90% CI) = 0.025 (0.024, 0.026)). Physical, psychosocial, and cognitive health were positively associated with life space mobility. Being less afraid to walk after dark (environmental variable) was also associated with greater mobility. Financial status influenced mobility through positive associations with psychosocial health and physical health. Higher education was related to better cognitive function and better financial status. Age was indirectly associated with life space mobility through its negative associations with financial status, cognition, and physical health. The same model can be used for males and females. The comprehensive mobility framework was verified with the large population-based CLSA dataset. Continued use of the framework is supported as it accurately portrays the complexity of factors that influence older adults’ life space mobility.

